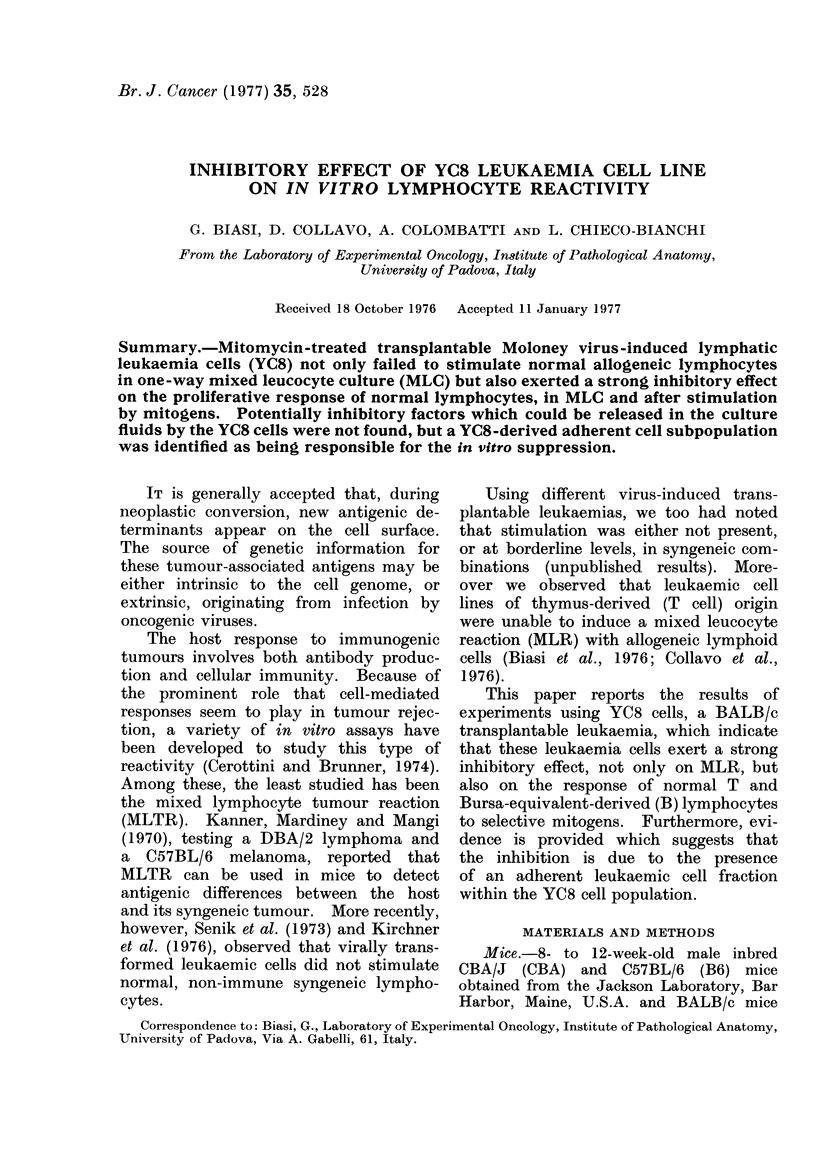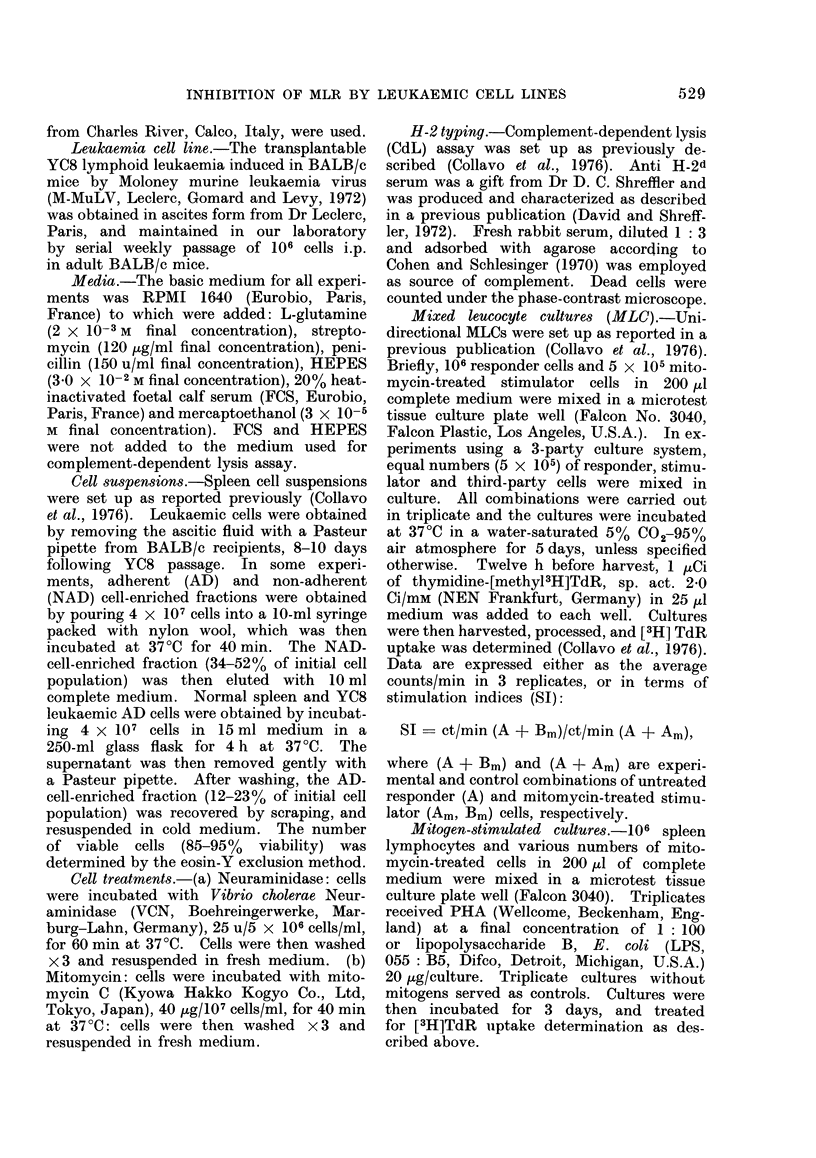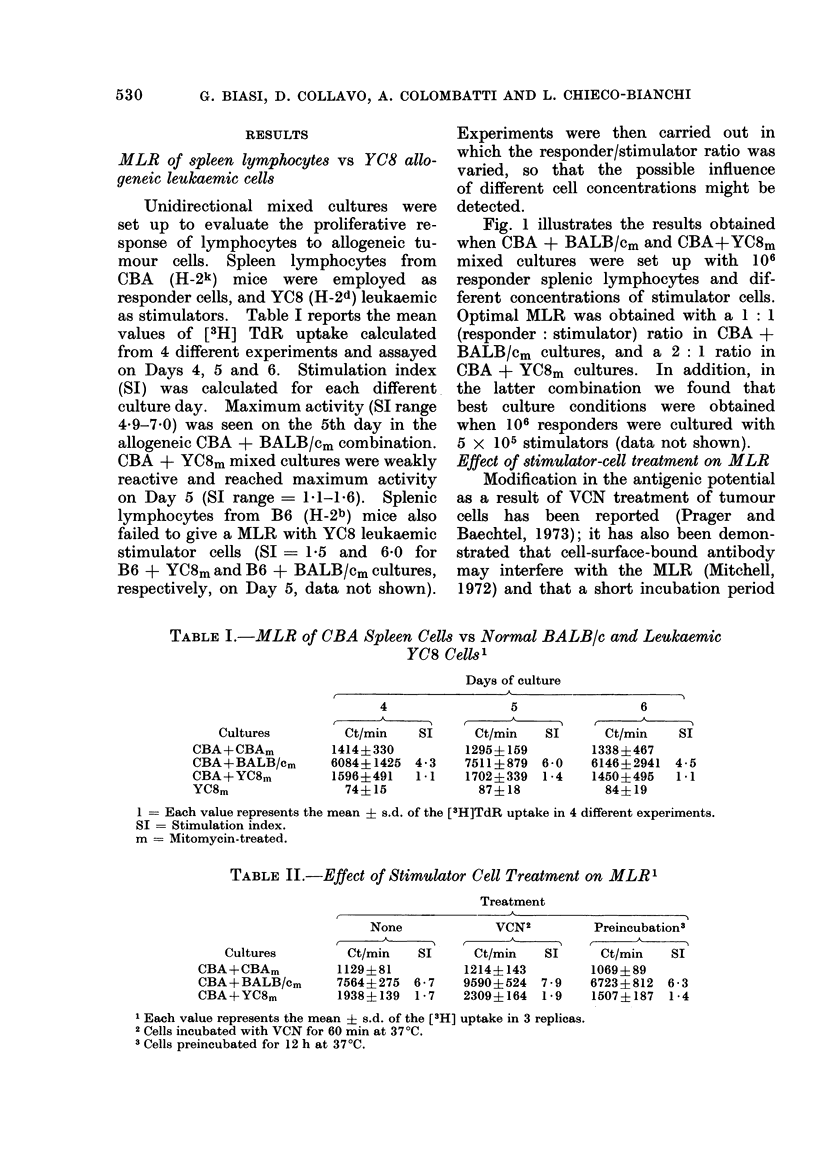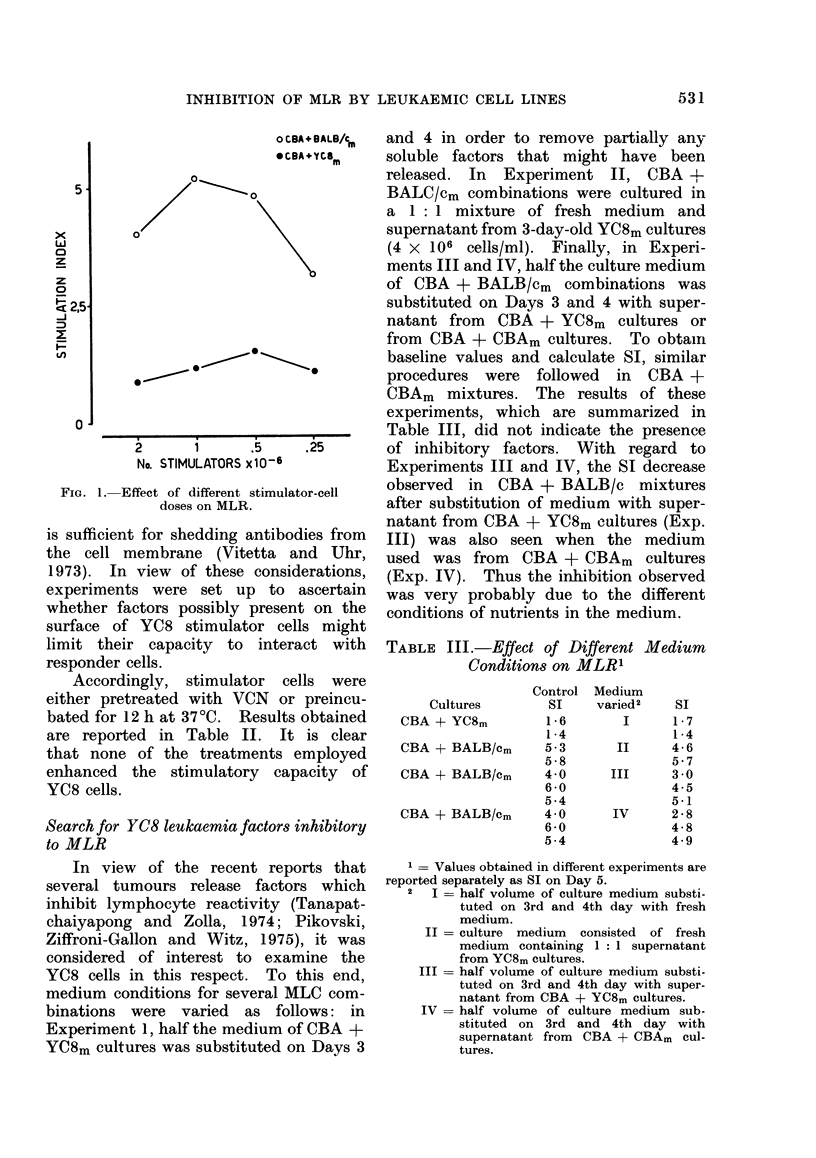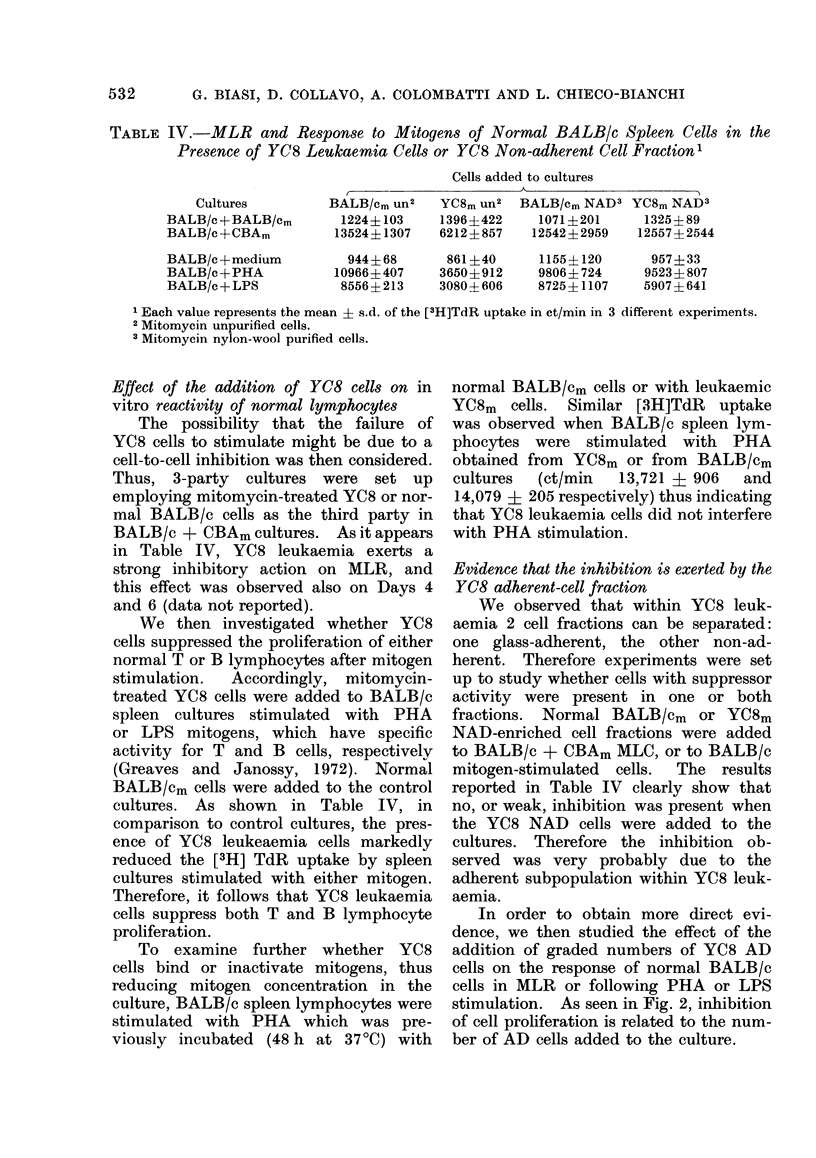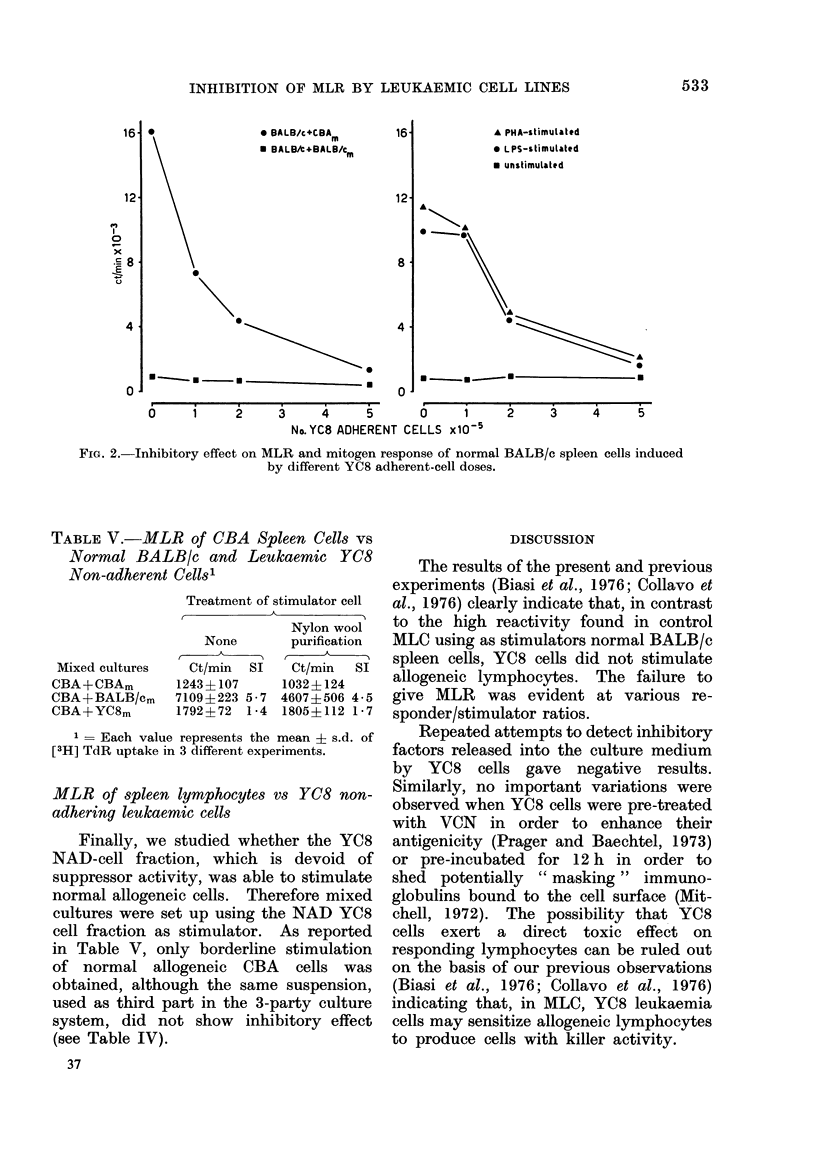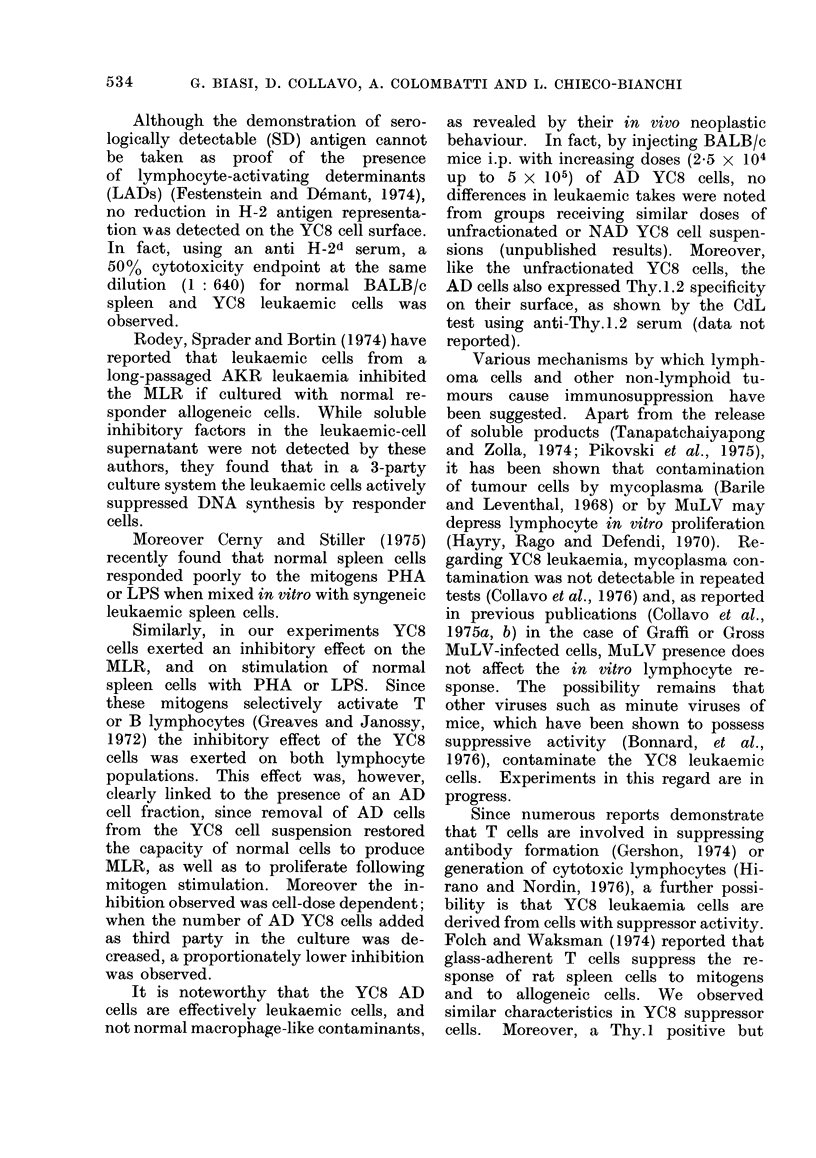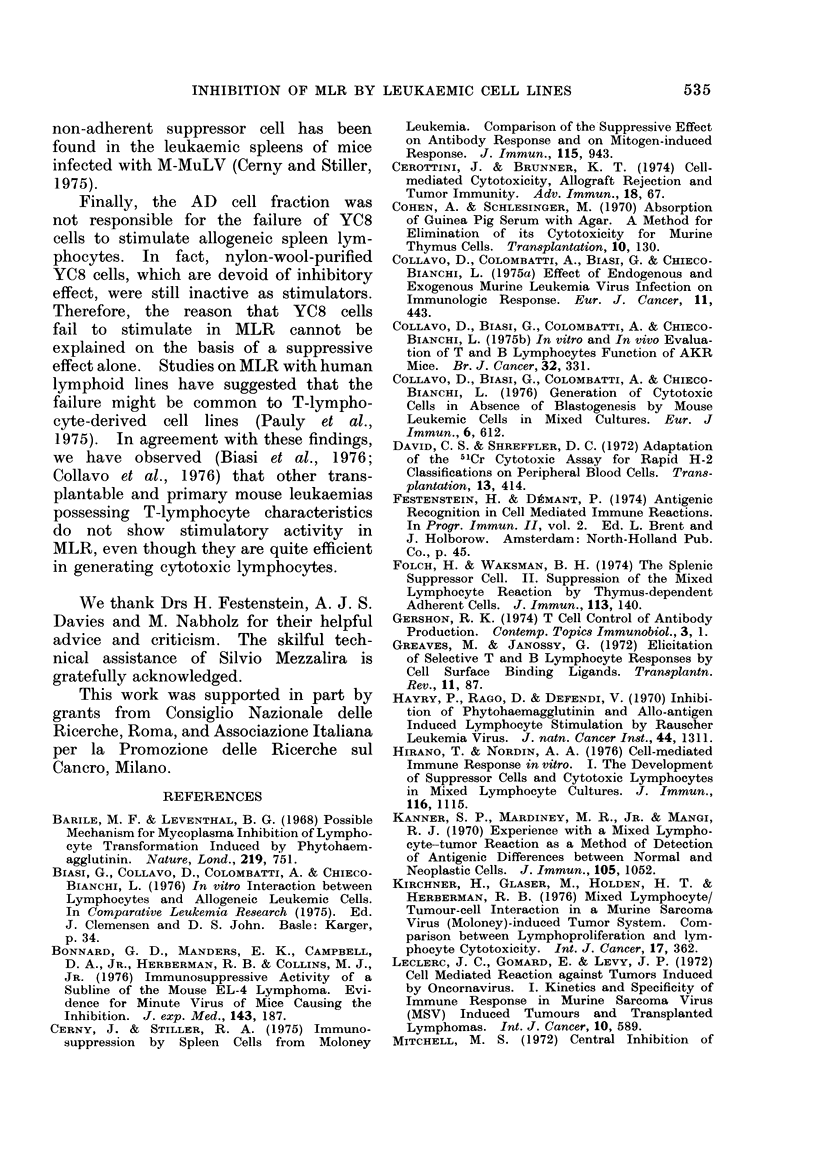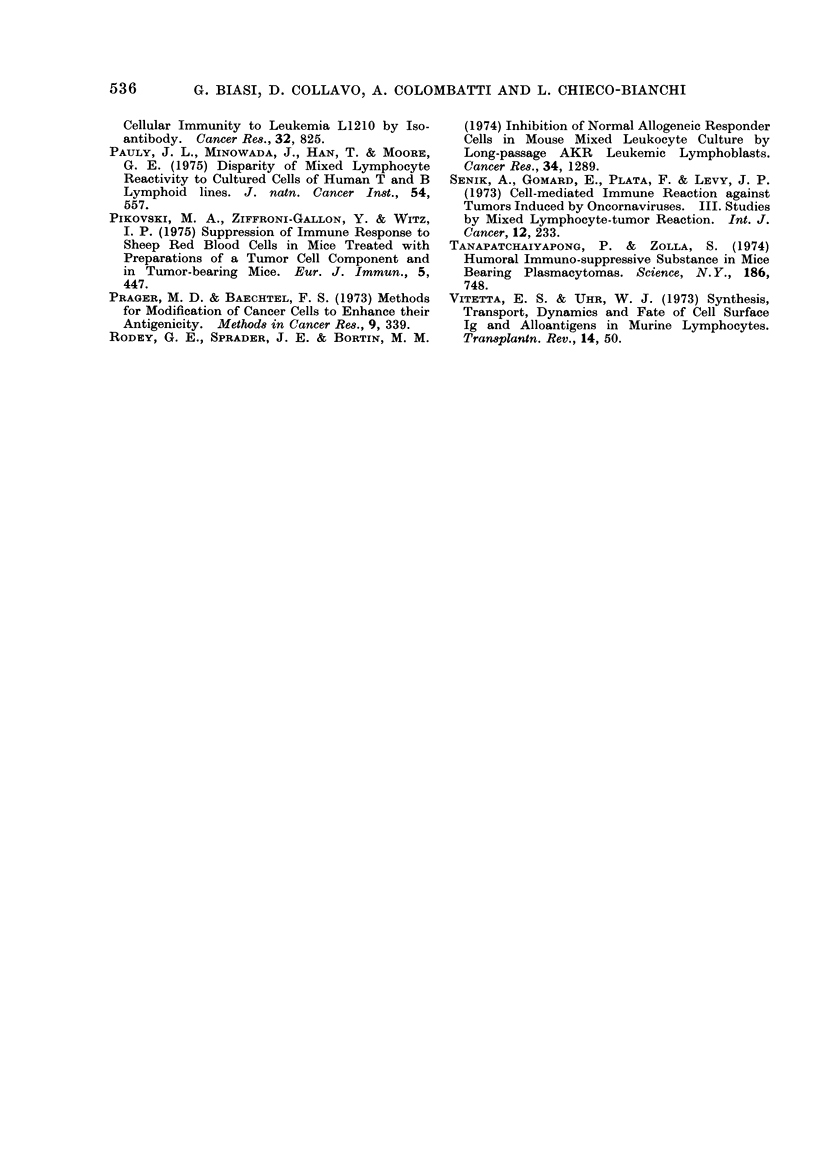# Inhibitory effect of YC8 leukaemia cell line on in vitro lymphocyte reactivity.

**DOI:** 10.1038/bjc.1977.86

**Published:** 1977-05

**Authors:** G. Biasi, D. Collavo, A. Colombatti, L. Chieco-Bianchi

## Abstract

Mitomycin-treated transplantable Moloney virus-induced lymphatic leukaemia cells (YC8) not only failed to stimulate normal allogeneic lymphocytes in one-way mixed leucocyte culture (MLC) but also exerted a strong inhibitory effect on the proliferative response of normal lymphocytes, in MLC and after stimulation by mitogens. Potentially inhibitory factors which could be released in the culture fluids by the YC8 cells were not found, but a YC8-derived adherent cell subpopulation was identified as being responsible for the in vitro suppression.


*Br. J. Cancer* (1977) **35**, 528

### INHIBITORY EFFECT OF YC8 LEUKAEMIA CELL LINE ON *IN VITRO* LYMPHOCYTE REACTIVITY

G. BIASI, D. COLLAVO, A. COLOMBATTI AND L. CHIECO-BIANCHI

*From the Laboratory of Experimental Oncology, Institute of Pathological Anatomy, University of Padova, Italy*

Received 18 October 1976  Accepted 11 January 1977

**Summary.—Mitomycin-treated transplantable Moloney virus-induced lymphatic leukaemia cells (YC8) not only failed to stimulate normal allogeneic lymphocytes in one-way mixed leucocyte culture (MLC) but also exerted a strong inhibitory effect on the proliferative response of normal lymphocytes, in MLC and after stimulation by mitogens. Potentially inhibitory factors which could be released in the culture fluids by the YC8 cells were not found, but a YC8-derived adherent cell subpopulation was identified as being responsible for the *in vitro* suppression.**

IT is generally accepted that, during neoplastic conversion, new antigenic determinants appear on the cell surface. The source of genetic information for these tumour-associated antigens may be either intrinsic to the cell genome, or extrinsic, originating from infection by oncogenic viruses.

The host response to immunogenic tumours involves both antibody production and cellular immunity. Because of the prominent role that cell-mediated responses seem to play in tumour rejection, a variety of *in vitro* assays have been developed to study this type of reactivity (Cerottini and Brunner, 1974). Among these, the least studied has been the mixed lymphocyte tumour reaction (MLTR). Kanner, Mardiney and Mangi (1970), testing a DBA/2 lymphoma and a C57BL/6 melanoma, reported that MLTR can be used in mice to detect antigenic differences between the host and its syngeneic tumour. More recently, however, Senik *et al.* (1973) and Kirchner *et al.* (1976), observed that virally transformed leukaemic cells did not stimulate normal, non-immune syngeneic lymphocytes.

Using different virus-induced transplantable leukaemias, we too had noted that stimulation was either not present, or at borderline levels, in syngeneic combinations (unpublished results). Moreover we observed that leukaemic cell lines of thymus-derived (T cell) origin were unable to induce a mixed leucocyte reaction (MLR) with allogeneic lymphoid cells (Biasi *et al.*, 1976; Collavo *et al.*, 1976).

This paper reports the results of experiments using YC8 cells, a BALB/c transplantable leukaemia, which indicate that these leukaemia cells exert a strong inhibitory effect, not only on MLR, but also on the response of normal T and Bursa-equivalent-derived (B) lymphocytes to selective mitogens. Furthermore, evidence is provided which suggests that the inhibition is due to the presence of an adherent leukaemic cell fraction within the YC8 cell population.

### MATERIALS AND METHODS

*Mice.*—8- to 12-week-old male inbred CBA/J (CBA) and C57BL/6 (B6) mice obtained from the Jackson Laboratory, Bar Harbor, Maine, U.S.A. and BALB/c mice

Correspondence to: Biasi, G., Laboratory of Experimental Oncology, Institute of Pathological Anatomy, University of Padova, Via A. Gabelli, 61, Italy.

from Charles River, Calco, Italy, were used.

*Leukaemia cell line.*—The transplantable YC8 lymphoid leukaemia induced in BALB/c mice by Moloney murine leukaemia virus (M-MuLV, Leclerc, Gomard and Levy, 1972) was obtained in ascites form from Dr Leclerc, Paris, and maintained in our laboratory by serial weekly passage of $10^6$ cells i.p. in adult BALB/c mice.

*Media.*—The basic medium for all experiments was RPMI 1640 (Eurobio, Paris, France) to which were added: L-glutamine ($2 \times 10^{-3}$ M final concentration), streptomycin (120 μg/ml final concentration), penicillin (150 u/ml final concentration), HEPES ($3{\cdot}0 \times 10^{-2}$ M final concentration), 20% heat-inactivated foetal calf serum (FCS, Eurobio, Paris, France) and mercaptoethanol ($3 \times 10^{-5}$ M final concentration). FCS and HEPES were not added to the medium used for complement-dependent lysis assay.

*Cell suspensions.*—Spleen cell suspensions were set up as reported previously (Collavo *et al.*, 1976). Leukaemic cells were obtained by removing the ascitic fluid with a Pasteur pipette from BALB/c recipients, 8–10 days following YC8 passage. In some experiments, adherent (AD) and non-adherent (NAD) cell-enriched fractions were obtained by pouring $4 \times 10^7$ cells into a 10-ml syringe packed with nylon wool, which was then incubated at 37°C for 40 min. The NAD-cell-enriched fraction (34–52% of initial cell population) was then eluted with 10 ml complete medium. Normal spleen and YC8 leukaemic AD cells were obtained by incubating $4 \times 10^7$ cells in 15 ml medium in a 250-ml glass flask for 4 h at 37°C. The supernatant was then removed gently with a Pasteur pipette. After washing, the AD-cell-enriched fraction (12–23% of initial cell population) was recovered by scraping, and resuspended in cold medium. The number of viable cells (85–95% viability) was determined by the eosin-Y exclusion method.

*Cell treatments.*—(a) Neuraminidase: cells were incubated with *Vibrio cholerae* Neuraminidase (VCN, Boehreingerwerke, Marburg–Lahn, Germany), 25 u/$5 \times 10^6$ cells/ml, for 60 min at 37°C. Cells were then washed ×3 and resuspended in fresh medium. (b) Mitomycin: cells were incubated with mitomycin C (Kyowa Hakko Kogyo Co., Ltd, Tokyo, Japan), 40 μg/$10^7$ cells/ml, for 40 min at 37°C: cells were then washed ×3 and resuspended in fresh medium.

*H-2 typing.*—Complement-dependent lysis (CdL) assay was set up as previously described (Collavo *et al.*, 1976). Anti H-$2^d$ serum was a gift from Dr D. C. Shreffler and was produced and characterized as described in a previous publication (David and Shreffler, 1972). Fresh rabbit serum, diluted 1 : 3 and adsorbed with agarose according to Cohen and Schlesinger (1970) was employed as source of complement. Dead cells were counted under the phase-contrast microscope.

*Mixed leucocyte cultures (MLC).*—Unidirectional MLCs were set up as reported in a previous publication (Collavo *et al.*, 1976). Briefly, $10^6$ responder cells and $5 \times 10^5$ mitomycin-treated stimulator cells in 200 μl complete medium were mixed in a microtest tissue culture plate well (Falcon No. 3040, Falcon Plastic, Los Angeles, U.S.A.). In experiments using a 3-party culture system, equal numbers ($5 \times 10^5$) of responder, stimulator and third-party cells were mixed in culture. All combinations were carried out in triplicate and the cultures were incubated at 37°C in a water-saturated 5% $CO_2$–95% air atmosphere for 5 days, unless specified otherwise. Twelve h before harvest, 1 μCi of thymidine-[methyl$^3$H]TdR, sp. act. 2·0 Ci/mM (NEN Frankfurt, Germany) in 25 μl medium was added to each well. Cultures were then harvested, processed, and [$^3$H] TdR uptake was determined (Collavo *et al.*, 1976). Data are expressed either as the average counts/min in 3 replicates, or in terms of stimulation indices (SI):

$$SI = ct/min\ (A + B_m)/ct/min\ (A + A_m),$$

where $(A + B_m)$ and $(A + A_m)$ are experimental and control combinations of untreated responder (A) and mitomycin-treated stimulator ($A_m$, $B_m$) cells, respectively.

*Mitogen-stimulated cultures.*—$10^6$ spleen lymphocytes and various numbers of mitomycin-treated cells in 200 μl of complete medium were mixed in a microtest tissue culture plate well (Falcon 3040). Triplicates received PHA (Wellcome, Beckenham, England) at a final concentration of 1 : 100 or lipopolysaccharide B, *E. coli* (LPS, 055 : B5, Difco, Detroit, Michigan, U.S.A.) 20 μg/culture. Triplicate cultures without mitogens served as controls. Cultures were then incubated for 3 days, and treated for [$^3$H]TdR uptake determination as described above.

### RESULTS

### *MLR of spleen lymphocytes* vs *YC8 allogeneic leukaemic cells*

Unidirectional mixed cultures were set up to evaluate the proliferative response of lymphocytes to allogeneic tumour cells. Spleen lymphocytes from CBA (H-$2^k$) mice were employed as responder cells, and YC8 (H-$2^d$) leukaemic as stimulators. Table I reports the mean values of [$^3$H] TdR uptake calculated from 4 different experiments and assayed on Days 4, 5 and 6. Stimulation index (SI) was calculated for each different culture day. Maximum activity (SI range 4·9–7·0) was seen on the 5th day in the allogeneic CBA + BALB/$c_m$ combination. CBA + $YC8_m$ mixed cultures were weakly reactive and reached maximum activity on Day 5 (SI range = 1·1–1·6). Splenic lymphocytes from B6 (H-$2^b$) mice also failed to give a MLR with YC8 leukaemic stimulator cells (SI = 1·5 and 6·0 for B6 + $YC8_m$ and B6 + BALB/$c_m$ cultures, respectively, on Day 5, data not shown). Experiments were then carried out in which the responder/stimulator ratio was varied, so that the possible influence of different cell concentrations might be detected.

Fig. 1 illustrates the results obtained when CBA + BALB/$c_m$ and CBA+$YC8_m$ mixed cultures were set up with $10^6$ responder splenic lymphocytes and different concentrations of stimulator cells. Optimal MLR was obtained with a 1 : 1 (responder : stimulator) ratio in CBA + BALB/$c_m$ cultures, and a 2 : 1 ratio in CBA + $YC8_m$ cultures. In addition, in the latter combination we found that best culture conditions were obtained when $10^6$ responders were cultured with $5 \times 10^5$ stimulators (data not shown).

### *Effect of stimulator-cell treatment on MLR*

Modification in the antigenic potential as a result of VCN treatment of tumour cells has been reported (Prager and Baechtel, 1973); it has also been demonstrated that cell-surface-bound antibody may interfere with the MLR (Mitchell, 1972) and that a short incubation period

TABLE I.—*MLR of CBA Spleen Cells* vs *Normal BALB/c and Leukaemic YC8 Cells*[1]

| | Days of culture | | | | | |
|---|---|---|---|---|---|---|
| | 4 | | 5 | | 6 | |
| Cultures | Ct/min | SI | Ct/min | SI | Ct/min | SI |
| CBA+$CBA_m$ | 1414±330 | | 1295±159 | | 1338±467 | |
| CBA+BALB/$c_m$ | 6084±1425 | 4·3 | 7511±879 | 6·0 | 6146±2941 | 4·5 |
| CBA+$YC8_m$ | 1596±491 | 1·1 | 1702±339 | 1·4 | 1450±495 | 1·1 |
| $YC8_m$ | 74±15 | | 87±18 | | 84±19 | |

1 = Each value represents the mean ± s.d. of the [$^3$H]TdR uptake in 4 different experiments.
SI = Stimulation index.
m = Mitomycin-treated.

TABLE II.—*Effect of Stimulator Cell Treatment on MLR*[1]

| | Treatment | | | | | |
|---|---|---|---|---|---|---|
| | None | | VCN[2] | | Preincubation[3] | |
| Cultures | Ct/min | SI | Ct/min | SI | Ct/min | SI |
| CBA+$CBA_m$ | 1129±81 | | 1214±143 | | 1069±89 | |
| CBA+BALB/$c_m$ | 7564±275 | 6·7 | 9590±524 | 7·9 | 6723±812 | 6·3 |
| CBA+$YC8_m$ | 1938±139 | 1·7 | 2309±164 | 1·9 | 1507±187 | 1·4 |

[1] Each value represents the mean ± s.d. of the [$^3$H] uptake in 3 replicas.
[2] Cells incubated with VCN for 60 min at 37°C.
[3] Cells preincubated for 12 h at 37°C.

FIG. 1.—Effect of different stimulator-cell doses on MLR.

is sufficient for shedding antibodies from the cell membrane (Vitetta and Uhr, 1973). In view of these considerations, experiments were set up to ascertain whether factors possibly present on the surface of YC8 stimulator cells might limit their capacity to interact with responder cells.

Accordingly, stimulator cells were either pretreated with VCN or preincubated for 12 h at 37°C. Results obtained are reported in Table II. It is clear that none of the treatments employed enhanced the stimulatory capacity of YC8 cells.

### *Search for YC8 leukaemia factors inhibitory to MLR*

In view of the recent reports that several tumours release factors which inhibit lymphocyte reactivity (Tanapatchaiyapong and Zolla, 1974; Pikovski, Ziffroni-Gallon and Witz, 1975), it was considered of interest to examine the YC8 cells in this respect. To this end, medium conditions for several MLC combinations were varied as follows: in Experiment 1, half the medium of CBA + $YC8_m$ cultures was substituted on Days 3 and 4 in order to remove partially any soluble factors that might have been released. In Experiment II, CBA + $BALC/c_m$ combinations were cultured in a 1 : 1 mixture of fresh medium and supernatant from 3-day-old $YC8_m$ cultures ($4 \times 10^6$ cells/ml). Finally, in Experiments III and IV, half the culture medium of CBA + $BALB/c_m$ combinations was substituted on Days 3 and 4 with supernatant from CBA + $YC8_m$ cultures or from CBA + $CBA_m$ cultures. To obtain baseline values and calculate SI, similar procedures were followed in CBA + $CBA_m$ mixtures. The results of these experiments, which are summarized in Table III, did not indicate the presence of inhibitory factors. With regard to Experiments III and IV, the SI decrease observed in CBA + BALB/c mixtures after substitution of medium with supernatant from CBA + $YC8_m$ cultures (Exp. III) was also seen when the medium used was from CBA + $CBA_m$ cultures (Exp. IV). Thus the inhibition observed was very probably due to the different conditions of nutrients in the medium.

TABLE III.—*Effect of Different Medium Conditions on MLR*[1]

| Cultures | Control SI | Medium varied[2] | SI |
|---|---|---|---|
| CBA + $YC8_m$ | 1·6 | I | 1·7 |
| | 1·4 | | 1·4 |
| CBA + $BALB/c_m$ | 5·3 | II | 4·6 |
| | 5·8 | | 5·7 |
| CBA + $BALB/c_m$ | 4·0 | III | 3·0 |
| | 6·0 | | 4·5 |
| | 5·4 | | 5·1 |
| CBA + $BALB/c_m$ | 4·0 | IV | 2·8 |
| | 6·0 | | 4·8 |
| | 5·4 | | 4·9 |

[1] = Values obtained in different experiments are reported separately as SI on Day 5.
[2] I = half volume of culture medium substituted on 3rd and 4th day with fresh medium.
II = culture medium consisted of fresh medium containing 1 : 1 supernatant from $YC8_m$ cultures.
III = half volume of culture medium substituted on 3rd and 4th day with supernatant from CBA + $YC8_m$ cultures.
IV = half volume of culture medium substituted on 3rd and 4th day with supernatant from CBA + $CBA_m$ cultures.

TABLE IV.—*MLR and Response to Mitogens of Normal BALB/c Spleen Cells in the Presence of YC8 Leukaemia Cells or YC8 Non-adherent Cell Fraction*[1]

| | Cells added to cultures | | | |
|---|---|---|---|---|
| Cultures | BALB/$c_m$ un[2] | $YC8_m$ un[2] | BALB/$c_m$ NAD[3] | $YC8_m$ NAD[3] |
| BALB/c+BALB/$c_m$ | $1224 \pm 103$ | $1396 \pm 422$ | $1071 \pm 201$ | $1325 \pm 89$ |
| BALB/c+$CBA_m$ | $13524 \pm 1307$ | $6212 \pm 857$ | $12542 \pm 2959$ | $12557 \pm 2544$ |
| BALB/c+medium | $944 \pm 68$ | $861 \pm 40$ | $1155 \pm 120$ | $957 \pm 33$ |
| BALB/c+PHA | $10966 \pm 407$ | $3650 \pm 912$ | $9806 \pm 724$ | $9523 \pm 807$ |
| BALB/c+LPS | $8556 \pm 213$ | $3080 \pm 606$ | $8725 \pm 1107$ | $5907 \pm 641$ |

[1] Each value represents the mean $\pm$ s.d. of the [$^3$H]TdR uptake in ct/min in 3 different experiments.
[2] Mitomycin unpurified cells.
[3] Mitomycin nylon-wool purified cells.

*Effect of the addition of YC8 cells on* in vitro *reactivity of normal lymphocytes*

The possibility that the failure of YC8 cells to stimulate might be due to a cell-to-cell inhibition was then considered. Thus, 3-party cultures were set up employing mitomycin-treated YC8 or normal BALB/c cells as the third party in BALB/c + $CBA_m$ cultures. As it appears in Table IV, YC8 leukaemia exerts a strong inhibitory action on MLR, and this effect was observed also on Days 4 and 6 (data not reported).

We then investigated whether YC8 cells suppressed the proliferation of either normal T or B lymphocytes after mitogen stimulation. Accordingly, mitomycin-treated YC8 cells were added to BALB/c spleen cultures stimulated with PHA or LPS mitogens, which have specific activity for T and B cells, respectively (Greaves and Janossy, 1972). Normal BALB/$c_m$ cells were added to the control cultures. As shown in Table IV, in comparison to control cultures, the presence of YC8 leukeaemia cells markedly reduced the [$^3$H] TdR uptake by spleen cultures stimulated with either mitogen. Therefore, it follows that YC8 leukaemia cells suppress both T and B lymphocyte proliferation.

To examine further whether YC8 cells bind or inactivate mitogens, thus reducing mitogen concentration in the culture, BALB/c spleen lymphocytes were stimulated with PHA which was previously incubated (48 h at 37°C) with normal BALB/$c_m$ cells or with leukaemic $YC8_m$ cells. Similar [3H]TdR uptake was observed when BALB/c spleen lymphocytes were stimulated with PHA obtained from $YC8_m$ or from BALB/$c_m$ cultures (ct/min 13,721 $\pm$ 906 and 14,079 $\pm$ 205 respectively) thus indicating that YC8 leukaemia cells did not interfere with PHA stimulation.

*Evidence that the inhibition is exerted by the YC8 adherent-cell fraction*

We observed that within YC8 leukaemia 2 cell fractions can be separated: one glass-adherent, the other non-adherent. Therefore experiments were set up to study whether cells with suppressor activity were present in one or both fractions. Normal BALB/$c_m$ or $YC8_m$ NAD-enriched cell fractions were added to BALB/c + $CBA_m$ MLC, or to BALB/c mitogen-stimulated cells. The results reported in Table IV clearly show that no, or weak, inhibition was present when the YC8 NAD cells were added to the cultures. Therefore the inhibition observed was very probably due to the adherent subpopulation within YC8 leukaemia.

In order to obtain more direct evidence, we then studied the effect of the addition of graded numbers of YC8 AD cells on the response of normal BALB/c cells in MLR or following PHA or LPS stimulation. As seen in Fig. 2, inhibition of cell proliferation is related to the number of AD cells added to the culture.

FIG. 2.—Inhibitory effect on MLR and mitogen response of normal BALB/c spleen cells induced by different YC8 adherent-cell doses.

TABLE V.—*MLR of CBA Spleen Cells* vs *Normal BALB/c and Leukaemic YC8 Non-adherent Cells*[1]

| | Treatment of stimulator cell | | | |
|---|---|---|---|---|
| | None | | Nylon wool purification | |
| Mixed cultures | Ct/min | SI | Ct/min | SI |
| CBA+CBA$_m$ | 1243±107 | | 1032±124 | |
| CBA+BALB/c$_m$ | 7109±223 | 5·7 | 4607±506 | 4·5 |
| CBA+YC8$_m$ | 1792±72 | 1·4 | 1805±112 | 1·7 |

[1] = Each value represents the mean ± s.d. of [$^3$H] TdR uptake in 3 different experiments.

*MLR of spleen lymphocytes vs YC8 non-adhering leukaemic cells*

Finally, we studied whether the YC8 NAD-cell fraction, which is devoid of suppressor activity, was able to stimulate normal allogeneic cells. Therefore mixed cultures were set up using the NAD YC8 cell fraction as stimulator. As reported in Table V, only borderline stimulation of normal allogeneic CBA cells was obtained, although the same suspension, used as third part in the 3-party culture system, did not show inhibitory effect (see Table IV).

### DISCUSSION

The results of the present and previous experiments (Biasi *et al.*, 1976; Collavo *et al.*, 1976) clearly indicate that, in contrast to the high reactivity found in control MLC using as stimulators normal BALB/c spleen cells, YC8 cells did not stimulate allogeneic lymphocytes. The failure to give MLR was evident at various responder/stimulator ratios.

Repeated attempts to detect inhibitory factors released into the culture medium by YC8 cells gave negative results. Similarly, no important variations were observed when YC8 cells were pre-treated with VCN in order to enhance their antigenicity (Prager and Baechtel, 1973) or pre-incubated for 12 h in order to shed potentially "masking" immunoglobulins bound to the cell surface (Mitchell, 1972). The possibility that YC8 cells exert a direct toxic effect on responding lymphocytes can be ruled out on the basis of our previous observations (Biasi *et al.*, 1976; Collavo *et al.*, 1976) indicating that, in MLC, YC8 leukaemia cells may sensitize allogeneic lymphocytes to produce cells with killer activity.

Although the demonstration of serologically detectable (SD) antigen cannot be taken as proof of the presence of lymphocyte-activating determinants (LADs) (Festenstein and Démant, 1974), no reduction in H-2 antigen representation was detected on the YC8 cell surface. In fact, using an anti H-$2^d$ serum, a 50% cytotoxicity endpoint at the same dilution (1 : 640) for normal BALB/c spleen and YC8 leukaemic cells was observed.

Rodey, Sprader and Bortin (1974) have reported that leukaemic cells from a long-passaged AKR leukaemia inhibited the MLR if cultured with normal responder allogeneic cells. While soluble inhibitory factors in the leukaemic-cell supernatant were not detected by these authors, they found that in a 3-party culture system the leukaemic cells actively suppressed DNA synthesis by responder cells.

Moreover Cerny and Stiller (1975) recently found that normal spleen cells responded poorly to the mitogens PHA or LPS when mixed *in vitro* with syngeneic leukaemic spleen cells.

Similarly, in our experiments YC8 cells exerted an inhibitory effect on the MLR, and on stimulation of normal spleen cells with PHA or LPS. Since these mitogens selectively activate T or B lymphocytes (Greaves and Janossy, 1972) the inhibitory effect of the YC8 cells was exerted on both lymphocyte populations. This effect was, however, clearly linked to the presence of an AD cell fraction, since removal of AD cells from the YC8 cell suspension restored the capacity of normal cells to produce MLR, as well as to proliferate following mitogen stimulation. Moreover the inhibition observed was cell-dose dependent; when the number of AD YC8 cells added as third party in the culture was decreased, a proportionately lower inhibition was observed.

It is noteworthy that the YC8 AD cells are effectively leukaemic cells, and not normal macrophage-like contaminants, as revealed by their *in vivo* neoplastic behaviour. In fact, by injecting BALB/c mice i.p. with increasing doses ($2{\cdot}5 \times 10^4$ up to $5 \times 10^5$) of AD YC8 cells, no differences in leukaemic takes were noted from groups receiving similar doses of unfractionated or NAD YC8 cell suspensions (unpublished results). Moreover, like the unfractionated YC8 cells, the AD cells also expressed Thy.1.2 specificity on their surface, as shown by the CdL test using anti-Thy.1.2 serum (data not reported).

Various mechanisms by which lymphoma cells and other non-lymphoid tumours cause immunosuppression have been suggested. Apart from the release of soluble products (Tanapatchaiyapong and Zolla, 1974; Pikovski *et al.*, 1975), it has been shown that contamination of tumour cells by mycoplasma (Barile and Leventhal, 1968) or by MuLV may depress lymphocyte *in vitro* proliferation (Hayry, Rago and Defendi, 1970). Regarding YC8 leukaemia, mycoplasma contamination was not detectable in repeated tests (Collavo *et al.*, 1976) and, as reported in previous publications (Collavo *et al.*, 1975*a*, *b*) in the case of Graffi or Gross MuLV-infected cells, MuLV presence does not affect the *in vitro* lymphocyte response. The possibility remains that other viruses such as minute viruses of mice, which have been shown to possess suppressive activity (Bonnard, *et al.*, 1976), contaminate the YC8 leukaemic cells. Experiments in this regard are in progress.

Since numerous reports demonstrate that T cells are involved in suppressing antibody formation (Gershon, 1974) or generation of cytotoxic lymphocytes (Hirano and Nordin, 1976), a further possibility is that YC8 leukaemia cells are derived from cells with suppressor activity. Folch and Waksman (1974) reported that glass-adherent T cells suppress the response of rat spleen cells to mitogens and to allogeneic cells. We observed similar characteristics in YC8 suppressor cells. Moreover, a Thy.1 positive but non-adherent suppressor cell has been found in the leukaemic spleens of mice infected with M-MuLV (Cerny and Stiller, 1975).

Finally, the AD cell fraction was not responsible for the failure of YC8 cells to stimulate allogeneic spleen lymphocytes. In fact, nylon-wool-purified YC8 cells, which are devoid of inhibitory effect, were still inactive as stimulators. Therefore, the reason that YC8 cells fail to stimulate in MLR cannot be explained on the basis of a suppressive effect alone. Studies on MLR with human lymphoid lines have suggested that the failure might be common to T-lymphocyte-derived cell lines (Pauly *et al.*, 1975). In agreement with these findings, we have observed (Biasi *et al.*, 1976; Collavo *et al.*, 1976) that other transplantable and primary mouse leukaemias possessing T-lymphocyte characteristics do not show stimulatory activity in MLR, even though they are quite efficient in generating cytotoxic lymphocytes.

We thank Drs H. Festenstein, A. J. S. Davies and M. Nabholz for their helpful advice and criticism. The skilful technical assistance of Silvio Mezzalira is gratefully acknowledged.

This work was supported in part by grants from Consiglio Nazionale delle Ricerche, Roma, and Associazione Italiana per la Promozione delle Ricerche sul Cancro, Milano.

### REFERENCES

Barile, M. F. & Leventhal, B. G. (1968) Possible Mechanism for Mycoplasma Inhibition of Lymphocyte Transformation Induced by Phytohaemagglutinin. *Nature, Lond.*, **219**, 751.

Biasi, G., Collavo, D., Colombatti, A. & Chieco-Bianchi, L. (1976) *In vitro* Interaction between Lymphocytes and Allogeneic Leukemic Cells. In *Comparative Leukemia Research* (1975). Ed. J. Clemensen and D. S. John. Basle: Karger, p. 34.

Bonnard, G. D., Manders, E. K., Campbell, D. A., Jr., Herberman, R. B. & Collins, M. J., Jr. (1976) Immunosuppressive Activity of a Subline of the Mouse EL-4 Lymphoma. Evidence for Minute Virus of Mice Causing the Inhibition. *J. exp. Med.*, **143**, 187.

Cerny, J. & Stiller, R. A. (1975) Immunosuppression by Spleen Cells from Moloney Leukemia. Comparison of the Suppressive Effect on Antibody Response and on Mitogen-induced Response. *J. Immun.*, **115**, 943.

Cerottini, J. & Brunner, K. T. (1974) Cell-mediated Cytotoxicity, Allograft Rejection and Tumor Immunity. *Adv. Immun.*, **18**, 67.

Cohen, A. & Schlesinger, M. (1970) Absorption of Guinea Pig Serum with Agar. A Method for Elimination of its Cytotoxicity for Murine Thymus Cells. *Transplantation*, **10**, 130.

Collavo, D., Colombatti, A., Biasi, G. & Chieco-Bianchi, L. (1975*a*) Effect of Endogenous and Exogenous Murine Leukemia Virus Infection on Immunologic Response. *Eur. J. Cancer*, **11**, 443.

Collavo, D., Biasi, G., Colombatti, A. & Chieco-Bianchi, L. (1975b) *In vitro* and *In vivo* Evaluation of T and B Lymphocytes Function of AKR Mice. *Br. J. Cancer*, **32**, 331.

Collavo, D., Biasi, G., Colombatti, A. & Chieco-Bianchi, L. (1976) Generation of Cytotoxic Cells in Absence of Blastogenesis by Mouse Leukemic Cells in Mixed Cultures. *Eur. J Immun.*, **6**, 612.

David, C. S. & Shreffler, D. C. (1972) Adaptation of the $^{51}Cr$ Cytotoxic Assay for Rapid H-2 Classifications on Peripheral Blood Cells. *Transplantation*, **13**, 414.

Festenstein, H. & Démant, P. (1974) Antigenic Recognition in Cell Mediated Immune Reactions. In *Progr. Immun. II*, vol. 2. Ed. L. Brent and J. Holborow. Amsterdam: North-Holland Pub. Co., p. 45.

Folch, H. & Waksman, B. H. (1974) The Splenic Suppressor Cell. II. Suppression of the Mixed Lymphocyte Reaction by Thymus-dependent Adherent Cells. *J. Immun.*, **113**, 140.

Gershon, R. K. (1974) T Cell Control of Antibody Production. *Contemp. Topics Immunobiol.*, **3**, 1.

Greaves, M. & Janossy, G. (1972) Elicitation of Selective T and B Lymphocyte Responses by Cell Surface Binding Ligands. *Transplantn. Rev.*, **11**, 87.

Hayry, P., Rago, D. & Defendi, V. (1970) Inhibition of Phytohaemagglutinin and Allo-antigen Induced Lymphocyte Stimulation by Rauscher Leukemia Virus. *J. natn. Cancer Inst.*, **44**, 1311.

Hirano, T. & Nordin, A. A. (1976) Cell-mediated Immune Response *in vitro*. I. The Development of Suppressor Cells and Cytotoxic Lymphocytes in Mixed Lymphocyte Cultures. *J. Immun.*, **116**, 1115.

Kanner, S. P., Mardiney, M. R., Jr. & Mangi, R. J. (1970) Experience with a Mixed Lymphocyte–tumor Reaction as a Method of Detection of Antigenic Differences between Normal and Neoplastic Cells. *J. Immun.*, **105**, 1052.

Kirchner, H., Glaser, M., Holden, H. T. & Herberman, R. B. (1976) Mixed Lymphocyte/Tumour-cell Interaction in a Murine Sarcoma Virus (Moloney)-induced Tumor System. Comparison between Lymphoproliferation and lymphocyte Cytotoxicity. *Int. J. Cancer*, **17**, 362.

Leclerc, J. C., Gomard, E. & Levy, J. P. (1972) Cell Mediated Reaction against Tumors Induced by Oncornavirus. I. Kinetics and Specificity of Immune Response in Murine Sarcoma Virus (MSV) Induced Tumours and Transplanted Lymphomas. *Int. J. Cancer*, **10**, 589.

Mitchell, M. S. (1972) Central Inhibition of

Cellular Immunity to Leukemia L1210 by Isoantibody. *Cancer Res.*, **32**, 825.

PAULY, J. L., MINOWADA, J., HAN, T. & MOORE, G. E. (1975) Disparity of Mixed Lymphocyte Reactivity to Cultured Cells of Human T and B Lymphoid lines. *J. natn. Cancer Inst.*, **54**, 557.

PIKOVSKI, M. A., ZIFFRONI-GALLON, Y. & WITZ, I. P. (1975) Suppression of Immune Response to Sheep Red Blood Cells in Mice Treated with Preparations of a Tumor Cell Component and in Tumor-bearing Mice. *Eur. J. Immun.*, **5**, 447.

PRAGER, M. D. & BAECHTEL, F. S. (1973) Methods for Modification of Cancer Cells to Enhance their Antigenicity. *Methods in Cancer Res.*, **9**, 339.

RODEY, G. E., SPRADER, J. E. & BORTIN, M. M. (1974) Inhibition of Normal Allogeneic Responder Cells in Mouse Mixed Leukocyte Culture by Long-passage AKR Leukemic Lymphoblasts. *Cancer Res.*, **34**, 1289.

SENIK, A., GOMARD, E., PLATA, F. & LEVY, J. P. (1973) Cell-mediated Immune Reaction against Tumors Induced by Oncornaviruses. III. Studies by Mixed Lymphocyte-tumor Reaction. *Int. J. Cancer*, **12**, 233.

TANAPATCHAIYAPONG, P. & ZOLLA, S. (1974) Humoral Immuno-suppressive Substance in Mice Bearing Plasmacytomas. *Science, N.Y.*, **186**, 748.

VITETTA, E. S. & UHR, W. J. (1973) Synthesis, Transport, Dynamics and Fate of Cell Surface Ig and Alloantigens in Murine Lymphocytes. *Transplantn. Rev.*, **14**, 50.